# Effects of Glucose and Vitamin D _3_ on Heat Stress Resistance in a *Caenorhabditis elegans* Model of Polyglutamine Aggregation Disease

**DOI:** 10.17912/micropub.biology.002376

**Published:** 2026-10-10

**Authors:** London Blackwell, Joseph Sharp, Gisselle Hernandez, Mindy Farris

**Affiliations:** 1 Biology, University of Central Arkansas; 2 Biology, University of Central Arkansas, Conway, AR, United States

## Abstract

Polyglutamine (polyQ) diseases are the most common form of inheritable neurodegeneration. Neuronal protein aggregates strongly correlate with polyQ symptoms; pathways that protect against protein aggregation may help mitigate the symptoms of poly-Q or delay disease onset. We used a *C. elegans* polyQ model, exposed to a heat stressor, to test potential protective mechanisms: glucose and vitamin D₃ (VD₃) supplementation. Glucose enrichment further shortened post-heat stress lifespan, while VD₃ in moderate doses trended toward restoring polyQ post-stress lifespan back to wild-type levels. These results show effects of early-life interventions that could indicate aspects of polyQ pathogenesis mechanisms and treatment options.

**Figure 1. Kaplan-Meier survival curve for C. elegans heat-stressed at 35ºC for 7 hours, at day 1 of adulthood, after overnight treatment with A) 250mM glucose (sorbitol control), or B-D) 400µM or 800µM Vitamin D 3 (ethanol control):** A) Wild-type ( N2 ) worms showed a 20.6% increase in post-heat stress lifespan with glucose (p=0.0234), but Q40 worms showed a 16.7% decrease in post-heat stress lifespan after glucose treatment (p=0.0229). B-D) Neither vitamin D _3_ concentration had a significant effect on post-heat stress lifespan for wild-type ( N2 ), Q24, or Q40 worms. There was a non-significant trend for 400µM to slightly lengthen Q40 post-heat stress lifespan (20.4%, p=0.0527). *The Q40 800µM VD3 line ends before 0 survival as the last worms in this condition died censored deaths.

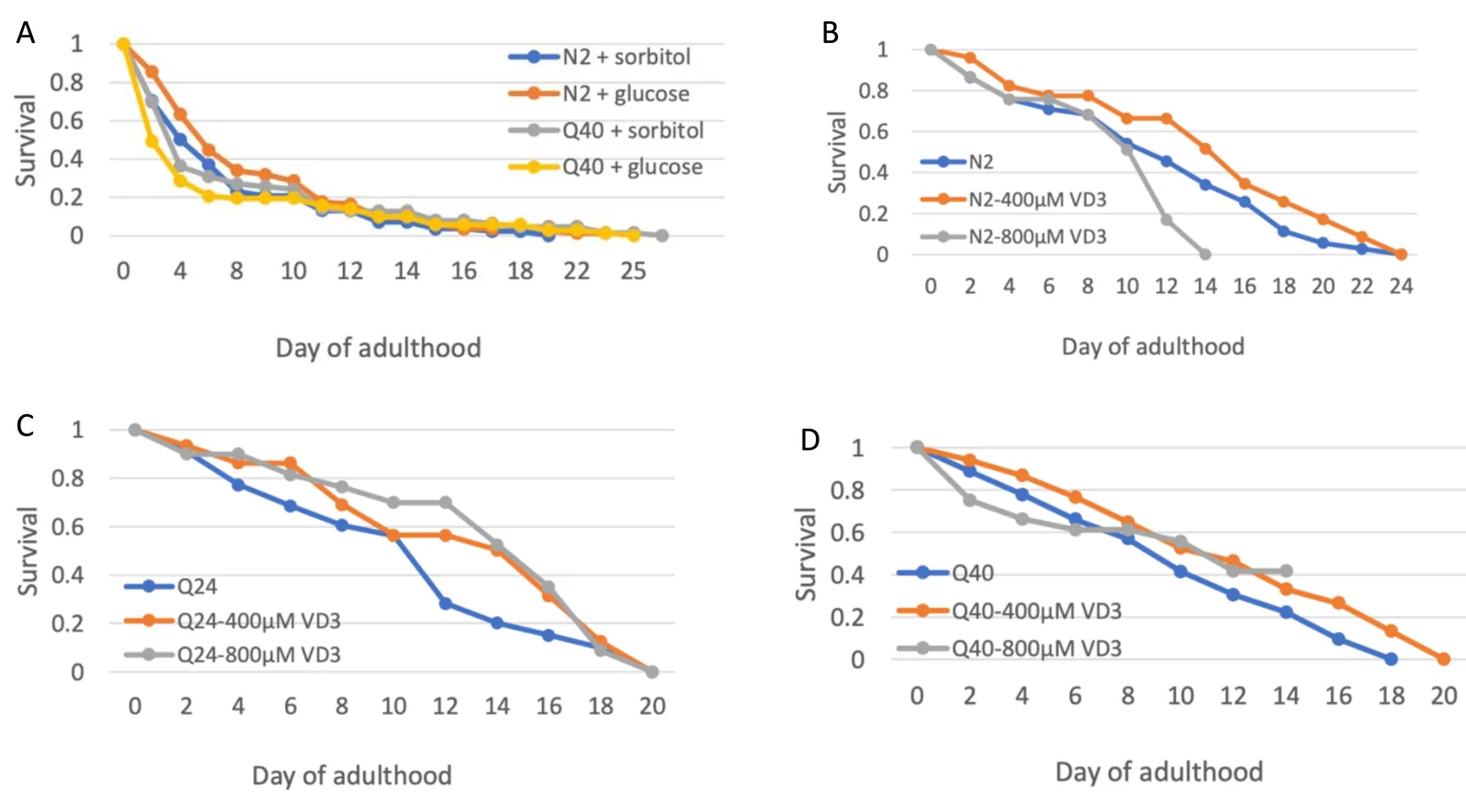



**Table:** 

Strain/treatment	Natural deaths	Censored deaths*	Median lifespan (days)	Mean lifespan (days) ± SE	p-value§
					
N2 sorbitol	114	31	6	6.3 ± 0.4	
N2 glucose	113	38	6	7.6 ± 0.4	0.0234
Q40 sorbitol	111	45	4	6.6 ± 0.6	
Q40 glucose	115	21	2	5.5 ± 0.5	0.0229
N2	46	42	12	11.6 ± 0.9	
N2 400µM VD	16	34	16	14.1 ± 1.5	0.0803
N2 800µM VD	17	20	12	9.5 ± 0.8	0.1776
Q24	42	56	12	10.5 ± 0.8	
Q24 400µM VD	19	30	16	12.8 ± 1.2	0.1669
Q24 800µM VD	16	24	16	13.5 ± 1.2	0.1138
Q40	47	42	10	9.8 ± 0.7	
Q40 400µM VD	34	18	12	11.8 ± 0.9	0.0527
Q40 800µM VD	16	24	12	8.4 ± 0.8	0.9451
*Worms that died of non-natural deaths, such as internal hatching of progeny, were censored when identified, but censored worms were included in the population up to this day.					
§Mean lifespans of worms treated with glucose are compared to mean lifespans of the same strain treated with sorbitol, used as an osmotic control. Mean lifespans of worms treated with VD3 are compared to mean lifespans of the same strain when untreated.					

## Description

Huntington's disease (HD) and eight other polyglutamine diseases are characterized by a CAG expansion in the nine causative genes, leading to a polyglutamine (polyQ) expansion in the respective resulting proteins, with longer poly-Q stretches (greater than 35Q in HD) leading to disease phenotypes, and the length of expansion inversely correlated to age-of-onset (Kikis et al., 2010; Pradhan et al., 2022; Weydt and La Spada, 2006). While *Caenorhabditis elegans* lack the *huntingtin* gene, *C. elegans* models of HD have shown protein aggregation and shortened lifespans conferred by expression of a transgenic polyQ stretch alone (Gidalevitz et al., 2006). This transgenic polyQ model provides a convenient system to study the mechanisms of polyQ pathogenesis and to test potential interventions.

Glucose enrichment typically reduces *C. elegans* lifespan, but can be protective at early ages, particularly against stressors like prolonged heat (Schulz et al., 2007; Smith et al., 2024). Dietary restriction (DR) protects against proteotoxic stress at least in some cellular backgrounds, but tissue of expression appears to play a role (Steinkraus et al., 2008; Tauffenberger et al., 2012). Studies in *C. elegans* HD models also show high concentrations of glucose could protect against polyQ proteotoxic stress, with tissue of expression important for efficacy (Gatrell et al., 2020; Tauffenberger et al., 2012). A strong link has been shown between longevity and resistance to various types of stressors: severe stress shortens lifespan, and many interventions that extend lifespan appear to also confer resistance to severe stressors (Kenyon, 2010). While the link between stress resistance and longevity is complex, stressors like heat exposure can exacerbate shortened lifespan phenotypes and mimic effects of age with regard to induction of protein misfolding; proteotoxic stress in particular can be simulated by increased temperature (Calderwood et al., 2009; Gidalevitz et al., 2006). Measurements of response to prolonged heat can thus be a highly predictive measure of longevity, particularly in an expanded polyQ (Q40) background.

Another pathway showing lifespan extension in wild-type ( N2 ) *C. elegans* and in *C. elegans* models of Alzheimer's disease (AD) is vitamin D _3_ (VD₃) supplementation, potentially through decreased nutrient signaling as a primary mechanism (Huggins and Farris, 2023; Leiteritz et al., 2021; Mark et al., 2016; Messing et al., 2013). We tested effects of glucose enrichment and of VD₃ supplementation on early-life resistance to a heat stressor in a *C. elegans* model of polyQ disease (Q40).

*Glucose effects on HD early-life stress resistance*

To investigate the effects of exposure to glucose on early-life stress resistance in a *C. elegans* model of HD, wild-type ( N2 ) worms and worms expressing a 40-glutamine stretch (Q40) under the control of a pan-neuronal promoter were utilized. Worms were transferred to 250mM glucose or 250mM sorbitol control plates on day 0 of adulthood (L4 stage), followed by a 7-hour heat stress at 35°C on day 1 of adulthood. Worms were scored for post-heat-stress lifespan.

The wild-type ( N2 ) worms showed a 20.6% increase in post-heat stress lifespan when exposed to glucose (p=0.0234). The Q40 worms, however, showed a 16.7% decrease in post-heat stress lifespan on glucose compared to the sorbitol osmotic control (p=0.0229) (Figure A, Table A).

*Vitamin D _3_ effects on HD early-life stress resistance*

We investigated the effects of exposure to vitamin D _3_ on early-life heat stress resistance for wild-type ( N2 ) worms and worms with pan-neuronal Q40 expression. We also examined VD _3_ effects on worms expressing a 24-glutamine stretch (Q24) under the control of a body-wall muscle promoter. As the Q24 strain is under the polyQ threshold for symptoms in 8 of the 9 polyQ diseases, at least in humans, the Q24 strain primarily served as another control strain. Polyglutamine stretches lead to length-dependent aggregation and cellular dysfunction in both neurons and body-wall muscle cells (Teschendorf and Link, 2009), although the different tissue of expression could certainly affect pathogenesis as well. Worms were transferred to 400µM or 800 µM VD₃ or ethanol control plates on day 0 of adulthood (L4 stage), followed by a 7-hour heat stress at 35°C on day 1 of adulthood. Worms were scored for post-heat-stress lifespan.

Neither VD _3_ concentration had a significant effect on post-heat stress lifespan in any of the worm strains (Table B). The sample sizes for the VD _3_ trials were low, so it is difficult to solidify conclusions; there was a non-significant trend, however, for 400µM VD _3_ to lengthen post-heat stress lifespans, particularly that of the Q40 strain (20.4%, p=0.0527) (Figure B-D, Table B).

*C. elegans* have several mechanisms to respond to high temperature environments, including the heat-shock transcription factor-1 ( HSF-1 ) and DAF-16 transcription factor, which upregulate systems responsible for maintaining cellular and protein homeostasis (Park et al., 2017). These functions require energy sources, which leads to the reasoning that additional glucose could allow them to function at a higher capacity to protect the organism from damage. Indeed, neurons have a high glucose requirement, particularly in an HD background, seen in the wasting phenotype of HD patients and transgenic models (Aditi et al., 2016; Tramutola et al., 2023). While we did find that high glucose conferred a benefit to young adult wild-type worms enduring heat stress exposure, as has been documented previously (Smith et al., 2024), we did not see a benefit to polyQ (Q40) worms from glucose enrichment at this level (250mM) or at this early age of adulthood.

Vitamin D _3_ , however, at the lower dose (400µM) did show a partial rescue of the Q40 worm post-heat stress lifespan, and improved post-heat stress lifespan for all strains, although not statistically significant. The higher dose of VD _3_ (800µM) likely crossed into toxicity.

These results show effects of glucose and VD _3_ interventions specifically early in the animal's lifespan. We have seen later-life interventions to have distinct effects (Smith et al., 2024), and are eager to test these during mid-life and late-life timepoints. Learning when these pathways are activated could uncover parts of the polyglutamine pathogenesis mechanism, as well as suggest treatment options.

## Methods

*C. elegans maintenance*

*C. elegans* strains (see below) were purchased from the Caenorhabditis Genetics Center (CGC). *Escherichia coli OP50* ( OP50 ) was also obtained from the CGC, and was propagated into liquid cultures grown in Luria Broth. For growth of worm populations, 250μL live OP50 was spotted onto Nematode Growth Media (NGM) plates (Brenner, S., 1974) and allowed to grow at least one night before use. All strains were maintained at 20°C and on standard NGM plates spotted with OP50 . Populations were synchronized by bleach preparation if stock strains were contaminated, otherwise, populations were synchronized by picking young adult worms onto fresh plates.

*Heat stress assay*

Heat stress response analyses were performed for N2 , Q24, and Q40 strains on 6cm plates of regular NGM spotted with 250µL OP50 bacteria and treated with either 250mM glucose (250mM sorbitol control) or 400µM/800µM Vitamin D _3_ (ethanol control). Worms were placed on the respective treatment 1 day before heat stress, at day 0 of adulthood (L4), 25 worms per plate. Worms were exposed to 35ºC heat stress for 7 hours, at day 1 of adulthood. All worms were hermaphrodites. No FUdR was used. Worms were transferred away from progeny and scored for response to touch every other day, until all worms were dead. Worms that died of non-natural deaths, such as internal hatching of progeny, were censored when identified, but censored worms were included in the population up to this day. Results represent the survival rate from a minimum of two biological replicates.

Kaplan-Meier curves were generated using JMP software, and the nonparametric test, log-rank (Mantel-Cox) was utilized to identify differences in mean lifespans.

## Reagents

*C. elegans strains*

**Table:** 

Strain	Genotype	Source
N2	wild-type	CGC
AM138 (Q24)	rmls130 [unc-54p::Q24::YFP], expresses in body-wall muscle cells	CGC
AM101 (Q40)	rmls110 [F25B3.3p::Q40::YFP], pan-neuronal expression	CGC
